# Role of a Brief Intensive Observation Area with a Dedicated Team of Doctors in the Management of Acute Heart Failure Patients: A Retrospective Observational Study

**DOI:** 10.3390/medicina56050251

**Published:** 2020-05-21

**Authors:** Gabriele Savioli, Iride Francesca Ceresa, Federica Manzoni, Giovanni Ricevuti, Maria Antonietta Bressan, Enrico Oddone

**Affiliations:** 1 Emergency Department, IRCCS Policlinico San Matteo, 27100 Pavia, Italy; irideceresa@gmail.com; 2 PhD School in Experimental Medicine, Department of Clinical-Surgical, Diagnostic and Pediatric Sciences, University of Pavia, Pavia 27100, Italy; 3 Clinical Epidemiology and Biometry Unit, IRCCS Policlinico San Matteo, 27100 Pavia, Italy; f.manzoni@smatteo.pv.it; 4 Former Professor of Geriatric and Emergency Medicine, University of Pavia, 27100 Pavia, Italy; giovanni.ricevuti@unipv.it; 5 Past Director Emergency Department, IRCCS Policlinico San Matteo, 27100 Pavia, Italy; mita.bressan@gmail.com; 6 Assistant Professor, Department of Public Health, Experimental and Forensic Medicine, University of Pavia, 27100 Pavia, Italy; enrico.oddone@unipv.it

**Keywords:** brief intensive observation (OBI), acute heart failure (AHF), emergency room, holding area, decision area

## Abstract

*Background and objectives*: Acute heart failure (AHF) is one of the main causes of hospitalization in Western countries. Usually, patients cannot be admitted directly to the wards (access block) and stay in the emergency room. Holding units are clinical decision units, or observation units, within the ED that are able to alleviate access block and to contribute to a reduction in hospitalization. Observation units have also been shown to play a role in specific clinical conditions, like the acute exacerbation of heart failure. This study aimed to analyze the impact of a brief intensive observation (OBI) area on the management of acute heart failure (AHF) patients. The OBI is a holding unit dedicated to the stabilization of unstable patients with a team of dedicated physicians. *Materials and Methods*: We conducted a retrospective and single-centered observational study with retrospective collection of the data of all patients who presented to our emergency department with AHF during 2017. We evaluated and compared two cohorts of patients, those treated in the OBI and those who were not, in terms of the reduction in color codes at discharge, mortality rate within the emergency room (ER), hospitalization rate, rate of transfer to less intensive facilities, and readmission rate at 7, 14, and 30 days after discharge. *Results*: We enrolled 920 patients from 1^st^ January to 31^st^ December. Of these, 61% were transferred to the OBI for stabilization. No statistically significant difference between the OBI and non-OBI populations in terms of age and gender was observed. OBI patients had worse clinical conditions on arrival. The patients treated in the OBI had longer process times, which would be expected, to allow patient stabilization. The stabilization rate in the OBI was higher, since presumably OBI admission protected patients from “worse condition” at discharge. *Conclusions:* Data from our study show that a dedicated area of the ER, such as the OBI, has progressively allowed a change in the treatment path of the patient, where the aim is no longer to admit the patient for processing but to treat the patient first and then, if necessary, admit or refer. This has resulted in very good feedback on patient stabilization and has resulted in a better management of beds, reduced admission rates, and reduced use of high intensity care beds.

## 1. Introduction

Acute heart failure (AHF) is one of the main causes of hospitalization in Western countries; it is estimated to account for about 1−2% of visits to the emergency department (ED) and the figure rises to more than 10% in patients over 70 years of age. Approximately 70−80% of ED patients with AHF have clinical indications for hospitalization [1,2]. AHF accounts for 5% of all causes of hospitalization for an acute episode, and 10% of hospitalized patients. It is responsible for about 2% of health expenditure, much of which is due to hospitalization costs. It is estimated that there will be a total mortality rate of 50% at 4 years. Among AHF patients, mortality and rehospitalization are 40% per year. In the last decade, international databases [3,4,5] show that AHF mainly affects the elderly, with an average age of 75, and that men and women are equally affected. Heart failure is a condition causing repeat acute care use. Heart failure patients have higher rates of readmission and ED revisitation than other patients. ED visits have increased dramatically in the last decade. However, little research has focused on emergency department (ED) visits. The ED not only plays an important role in returning patients after an inpatient discharge, but can also prevent the need for a longer inpatient stay for well-timed visits. Recent studies have shown that current methods of measuring hospital readmissions focus only on inpatient-to-inpatient hospitalization and ignore return visits to the emergency department (ED) that do not result in an admission. The relative importance of the return ED visit is currently not well established. However, current hospital readmission measures focus only on repeat inpatient care episodes, overlooking patients who return for care to the ED, but were not actually admitted. Some studies suggest that nearly half of all 30-day return visits from an inpatient stay might be missed by focusing only on patients who are readmitted. Other studies show that approximately one in five patients are presented to the ED within 30 days of an inpatient hospitalization and over half of these patients were readmitted. Current efforts to identify patients at risk of repeat acute care use must therefore also take into account ED visits. Our study focuses on all admissions of patients with AHF to acute care, analyzing and focusing above all on the “gray part” that will not be hospitalized, previously neglected by other studies [6,7,8,9,10,11,12,13,14,15].

Usually, patients cannot be admitted directly to the wards. The optimal organization and management of the emergency room (ER) is therefore essential for the effective management of acute pathologies, and AHF in particular. Holding areas were born as a response to the phenomena of “access block” and “boarding”. Access block refers to the delay in patients gaining access to inpatient beds after being admitted [16,17,18,19,20]. Numerous studies from the US, UK, Canada, and Australia have shown that access block causes ED overcrowding and affects the quality of care. Within emergency medicine, many believe that the “boarding” of emergency department (ED) patients awaiting inpatient beds compromises the quality of care [20,21,22,23,24,25,26]. Holding units are clinical decision units, or observation units, within the ED. In the US, reviews by the Institute of Medicine Committee found that such units were able to alleviate access block and ED overcrowding. They also contribute to a reduction in hospitalization and improvements in ambulatory care [27,28,29]. Observation units have also been shown to play a role in specific clinical conditions, like the acute exacerbation of heart failure, which is a very common cause for hospital admission [30,31,32,33,34,35]. Some studies, on the other hand, have shown only small improvements after adopting decision units (reduced ED length of stay, reduced admission rate, and no increase in ED revisit rate) [32]. In summary, there is some evidence for the role of holding units for alleviating access block and overcrowding in the ED, but this must be implemented with carefully planned clinical management protocols and adequate support staff [20].

In our ER, AHF is a frequent reason for patient visits and admission. In response to the problem of access block, it was decided to set up a team of capable and experienced physicians to form the decision unit of our ER and be dedicated to the holding area, called brief intensive observation (OBI).

This study aimed to investigate whether OBI admission was associated with a significantly higher rate of patient stabilization, a lower percentage of transfers to other hospital wards or departments, and a lower percentage of hospitalizations.

The primary objective of the study was to investigate whether the AHF patients admitted to the OBI had a reduction in color codes at discharge compared to AHF patients not admitted to the OBI. The secondary objectives were to compare the following secondary endpoints between the two groups (OBI patients versus non-OBI patients): mortality rate within the ED, hospitalization rate, transfer rate to less intensive facilities, and readmission rate at 7, 14, and 30 days after discharge.

## 2. Materials and Methods

### 2.1. Overall Design

Eligibility criteria: Adult patients (≥18 years of age) who accessed the emergency department of San Matteo Hospital Foundation, Pavia, Italy, for AHF between 1^st^ January and 31^st^ December, 2017, with a state of consciousness not altered, ability to read, and consent to the processing of data for health and research purposes. Patients were assigned to the OBI group or the non-OBI group through a clinical evaluation which aimed to include those who were in a worse clinical condition in the first group. Patients who had a clear picture of AHF and needed intravenous therapy, non-invasive ventilation, C-PAP, cardioactive therapy (including amine), or continuous monitoring of vital parameters were sent to the OBI. Patients with the need to complete a differential diagnosis were also sent, which was thought to take more than 6 h. This group frequently includes patients who were suspected of having an acute ischemic disease of the NSTEMI type associated with the heart failure framework. Moreover, a further criterion of inclusion considered patients clinically judged to need hospitalization in the medical department but who were not yet stable hemodynamically (low-range imbalances frankly hypothesized, with hypertensive emergencies in progress). Patients in need of hospitalization for which a bed in the ward was not quickly available were sent also to the OBI. Additionally, patients were excluded due to peri-arrests or ACC with ongoing resuscitation maneuvers.

### 2.2. Study Design

This was a prospective single-center observational study with retrospective data collection through the software PiEsse.

The reduction in color codes at discharge was used as a suitable proxy for the degree of patient stabilization, our primary outcome, while the mortality rate within the ED, hospitalization rate, rate of transfer to less intensive care facilities, and readmission rate at 7, 14, and 30 days after discharge were considered as secondary outcomes.

Data were provided directly by the San Matteo Hospital Foundation, which keeps the files regarding all services that are provided by its ED. An ad hoc query was performed to obtain the data of interest. The first name and surname of patients were substituted with an anonymous code which ensured that the researchers were blind to the patient identities.

The data collection was retrospective; at the time of admission to the ER of the San Matteo Hospital Foundation the patient provided informed consent for the processing of data for medical and research purposes.

### 2.3. Statistical Analysis

Statistical analyses were carried out using appropriate logistic, univariate, and multivariate regression models to test the association between the assignment to the OBI group and clinical stabilization (reduction in color codes at discharge). Continuous variables were described as mean and standard deviation, while qualitative variables were expressed with counts and percentages.

Comparisons between the two groups of continuous variables were made with Student’s *t*-tests, while associations between the qualitative variables were studied with χ^2^ tests or Fisher’s exact tests when the number of observations within at least a single cell was equal to or lower than five.

The significance level was set at alpha 0.05 (statistical significance at *p*-value < 0.05) and all tests were two-tailed. The analyses were conducted with STATA software, version 14 (Stata Corporation, College Station, TX, USA, 2015).

## 3. Results

This study involved 920 consecutive patients who accessed the ED of San Matteo Hospital Foundation for AHF. Patients were equally divided between males (461, 50.11%) and females (459, 49.89%). The mean age was 78.3 years and 82.0 years for men and women, respectively, and the difference was statistically significant (*p* < 0.001). No other variable, such as arrhythmia, heart rate (HR), systolic and diastolic blood pressure (SBP, DBP), arterial oxygen saturation (SatO2), priority code at access, priority code at discharge, wait time, process time, or LOS, showed a significant difference between men and women.

A total of 562 (61.09%) of AHF patients were included in the OBI group, while 358 (38.91%) were in the non-OBI group. The main features of the two groups are reported in Table 1. Men within the OBI group showed a significantly higher mean age compared to men in the non-OBI group. There were no significant differences between the groups for vital signs, except for a higher mean HR for male patients in the OBI group.

The OBI group patients had worse clinical conditions on arrival, as indicated by a significantly higher percentage of “yellow” and “red” codes (*p* = 0.004), and, by contrast, a better clinical status at discharge with a lower percentage of “red” codes, compared to the non-OBI group (*p* = 0.001). Patients in the OBI group had a significantly (*p* = 0.029) lower mean wait time (50.6 min) compared to the non-OBI group (59.6 min), as well as a longer process time (mean: 606.1 min vs. 327.6 min; *p* < 0.001) and a longer length of stay (625.3 min vs. 377.0 min; *p* < 0.001). Length of stay is defined as the duration of the stay in the emergency room, including waiting for the medical examination, the duration of the process, and the phenomenon of boarding. No difference in mortality rate was observed between the two groups, while the OBI group had a significantly higher percentage of transfers to other hospital wards or departments and a significantly lower percentage of hospitalizations. This result was also confirmed when we adjusted for all potential confounding variables. No significant differences were observed regarding patients’ readmission at 7, 14, and 30 days after discharge (Table 2).

Finally, both univariate and multivariate logistic regression models show that being included in the OBI group significantly (*p* = 0.002) protected patients from being classified as “worse condition” at discharge, if this condition is taken as “red code” at discharge. Additionally, a longer wait time seems to play a minimal protective role, while higher HR values provide a small increase in risk (Table 3).

## 4. Discussion

Our ER is divided into areas dedicated to specific intensities of care. There is an area of low intensity and an area of medium–high intensity. Patients who arrive at our ED are first subjected to triage where specialized nurses with basic and advanced business training collect information related to the patient’s general data, the main presenting symptoms, and a short history. They then proceed to the measurement of vital signs and conduct a visual inspection. At this stage, based on written protocols (“triage grids”), drawn up mainly based on the evolution of the main symptoms, the patient’s medical history, and vital signs, the patients are assigned a priority code for the medical examination and are directed to an area of appropriate intensity of care.

There are five levels of priority code for the medical examination in our ED:

(a) Red code: immediate entry into the shock room (high-intensity area). It is assigned to patients with severe impairment of vital signs or consciousness.
(b) Yellow code with medium care intensity: immediate, or at least within 40 min, entry to the average intensity care area.
(c) Yellow code with low care intensity: immediate entry, or at least within 40 min, to the low intensity care area.
(d) Green code: assigned to deferred urgency or minor emergencies with a wait of a few hours and entry to the low intensity of care area.
(e) White code: non-urgent cases with a wait of a few hours and entry to the low intensity of care area.

The criteria for assigning a patient to the medium–high intensity care area include the deterioration of a vital sign or consciousness, the worsening of any concomitant symptoms (e.g., typical chest pain), the need for care, e.g., oxygen, or the need for multi-parameter monitoring.

The patient is then seen by the ER doctor who will set the patient’s therapeutic and diagnostic pathway. The two different areas of intensity of care converge on the stabilization area which is the OBI. The doctors in the room can use their clinical judgment to admit the patient directly without going to the OBI. At the end of the process, patients are admitted, discharged, or transferred to a hospital with a lower intensity of care depending on the degree of illness severity and the stabilization achieved. The patient’s condition on discharge or referral is categorized by the doctor with a color code. A red code is given to unstable patients, a yellow code to patients who are stabilized but still in need of medium-intensity care, and a green code to patients who are stabilized and still in need of low intensity care.

At the end of 2016, a team of doctors from our ED team was chosen to join the OBI team. The OBI team had as its mission the safe discharge or appropriate admission of patients, and to assist with the bed management of all emergency admissions. Because of the boarding and overcrowding, the need to develop an area in which to stabilize acute patients had become urgent [20,21,22,23,24,25,26,27,28]. Our hospital had no emergency medicine or stabilization area. It was decided to use the OBI team for this purpose because it was already functioning and it consisted of a small pool of doctors who had developed a closeness and homogeneity of patient management [29,30,31,32,33,36,37,38]. Given the wide range and complexity of patients and the complexity of bed management (with vacancies arising from various departments throughout the day) in a second-level ED, it was decided to draw up 12-h shifts, to create a more continuous and homogeneous service. In the other ED rooms, 6.5-h shifts were worked to avoid the well-known phenomenon of the deteriorating performance of doctors. From an organizational point of view, the OBI team was responsible for the management of beds for acute admissions. Their clinical duties included the management of the patients sent either by the different intensity care areas after an initial evaluation by the doctor in that area, or they could take a patient directly from the waiting room in case of overcrowding. They also managed patients in boarding and they stabilized complex patients who needed an average intensity of care. They assessed the functional capacity of patients, to assist with making clinical decisions and determining the need for home support for patients who were to be discharged home, and they carefully assessed and differentiated high-risk patients, who needed hospitalization, from low-risk patients.

Patients were managed in the OBI, an area of medium-intensity care; upon entry, they underwent a reassessment and had ECGs and laboratory tests if required, they also had the diagnostic process completed with first- or second-level imaging, if needed. A therapy sheet would immediately be drawn up so that the patient would continue on their existing drug therapy while avoiding polytherapy. Management in a medium-intensity area also allowed the close multi-parameter monitoring of patients. From the outset, this proved extremely beneficial for the patient, because in an acute setting “time is life”, and this type of system combined the regular and timely application of all the treatment that the patient needed, combined with close monitoring on the same emergency platform as the ER [30,31].

In our experience, this management model has shortened waiting times, improved the appropriateness of admissions, optimized the management of available health resources, and allowed better management of complex and serious patients that often crowd EDs, allowing them to be stabilized.

### 4.1. Evaluation of Our Experience

We analyzed the impact of the OBI team in the treatment of AHF. The majority of emergencies on medical examination are directed to the OBI, while patients with less urgent conditions are more often managed in the other ED areas. This is because patients with a greater need of stabilization were sent to the dedicated area that was there to manage a large proportion of the most complex patients. These data are in line with those in the international literature, as stable patients with low-risk AHF are usually managed in the ED and discharged home [33,34,35,36,37,38].

Process and LOS times were much higher, as expected [33,34,35,36,37,38], for patient stabilization. Achieving stabilization requires more process time with longer stays in the ER, but this allowed a reduction in adverse events and better management of available health resources and valuable beds.

Although mortality was reduced, it was not statistically significant. To confirm this, we believe that a wider cohort of patients need to be recruited. However, some studies have reported that mortality was similar when patients who were managed in an observation unit were compared to those who were admitted directly from the ED [35].

However, the degree of stabilization of that patients achieved was significantly higher, as demonstrated by the discharge code and the higher rate of transfer to hospitals with lower care intensity. This figure is in line with some studies that showed that admission to an observation unit reduced the rates of return to the ED with AHF, and admissions to both the observation unit and inpatient unit for AHF at 90 days [38]. Other studies have suggested that a specialized AHF observation unit may be best for patient care while reducing admission rates [38]. It has been found that observation units provide a cost-effective alternative, compared to hospital admission, for those with non-high-risk AHF (Acute Hear Failure) [30] by avoiding ordinary hospitalization. In more detail, we can summarize how therapies performed in the OBI (C-PAP, EV therapy, endovenous diuretics, cardioactive therapies) over a longer period of time have allowed some patients, at low to medium risk, to regain a AHF (Acute Hear Failure) compatible with home care. These patients, after acute therapy in the OBI, were given modified home therapy, and patients were then redirected with a facilitated pathway to outpatient care. The most unstable and high-risk patients were still hospitalized, but after appropriate stabilization, as already stated. An increased use of low-intensity hospital beds and a higher rate of transfer to hospitals with less intensity care has made the best use of resources.

Above, we have seen how patients stabilized at a level compatible with home care were redirected with a facilitated pathway to outpatient care with enhanced or modified home therapy. We have also seen that patients managed in the OBI have a higher stabilization rate and a lower rate of hospitalization.

The criteria for remissibility are the improvement of the imaging framework (with chest and/or cardiac ultrasound or with chest Rx), clinical signs (reduction of dependent edema), instrumental indices (hemogasanalysis, oxygen saturation), and patient symptoms. The lower rate of re-entry and ED visits in the group of patients treated in the OBI in the face of a higher rate of discharge, although not statistically significant, highlights the safety of discharge. Patients for whom outpatient care and the new home therapy were not sufficient showed readmission and ED revisitation. With regard to the data of readmission and ED revisitation, it must be specified that they are made more solid by the fact that our ER is the only one in our municipality, and we are the reference center of our province.

In our opinion, this is due to the well-established fact that immediately availing the patient of the prescribed acute therapy at the right time, and with the maintenance of home therapy, means regaining a period of treatment that could otherwise be lost. This may be because the hand-over of the patients to the duty staff may not guarantee the optimal timing of emergency therapy, or because delays due to overcrowding may interrupt the normal administration of the home therapy. Furthermore, some types of drugs may not be normally stocked in the ER. Seeing the evolution and response of the patient to therapy over time allows a better stratification of the risk. The longer process time also allows the patient to be monitored with cardiac and chest ultrasound to allow a careful assessment of risk and stabilization.

The greater degree of patient stabilization brings the great advantage of a more marked use of beds in low intensity wards and the increased transfer to outlying hospitals with lower levels of intensity of care [28,29,30].

The reduction in patients who left before being seen is usually interpreted as a patient satisfaction index and an indicator of good functioning of the ER. This, in our opinion, could be mainly due to the overall management of the patient and the degree of rapid stabilization. However, the presence of a physical area dedicated to the treatment of these patients, equipped with a bathroom and comfortable beds (the same as the wards), bedside tables, and so on, may also play a role. All this creates a more comfortable environment than other areas of the emergency room and can therefore result in better patient satisfaction and a consequent reduction in patients who leave before being seen. We believe that, above all, the presence of comfortable beds compared to stretchers can, especially in elderly patients, increase compliance with care.

The readmission rate was lower for patients managed in the OBI but was not statistically significant. This, too, may depend on nuance, because, as stated, some studies with larger cohorts have reported an advantage in terms of returning patients. However, it should also be noted, as some have reported, that the outcomes of 30-day readmission and recurrent ED visits for AHF or mortality were similar when patients managed in an observation unit were compared to those who were hospitalized directly from the ED [29].

### 4.2. Future Perspective

This model can be applied in situations, such as ours, where there is a limited availability of medium-intensity care beds in the hospital. For the best outcomes and the best management of available health resources, we propose a model in which a dedicated team, perhaps rotating, takes care of both the stabilization of complex patients and their admission, together with appropriate bed management.

### 4.3. Limitation

First, our conclusions are limited by the observational nature of the study, including the partly retrospective retrieval of information. Second, we did not compare the care patients received. Our outcomes may therefore have been affected by differing correctness or timeliness of the treatment. Another limitation of the study is that we do not have an echocardiographic or biochemical stratification of patients with heart failure. We therefore do not know whether the results are worth, for example, more for a heart failure with a major systolic or diastolic component. We also point out that a true shared typing of acute heart failure is not yet defined and that many international studies have begun to do so. However, we do not believe that this data affect the conclusions, as the advantage of this observational study is that it analyzes the real life of our emergency room.

## 5. Conclusions

A dedicated area of the ED, such as the OBI, may progressively allow us to change the processing of AHF patients with the aim of no longer admitting the patient for definitive processing, but to process and treat the patient and thereafter determine hospitalization. We achieved good results on patient stabilization. We also observed better management of beds, reduced admission rates, and reduced use of high-intensity beds. Limitations of the data recorded in the trust electronic records may affect the conclusions, we did not, for example, assess the prevalence of some comorbidities, such as COPD, chronic ischemic heart disease, or stroke.

## Figures and Tables

**Table 1:** Principal clinical and process features of OBI and non-OBI groups.

	OBI N (%)	Mean (95% IC)	Non-OBI N (%)	Mean (95% CI)	*p*
**Sex**					
Men	283 (50.4%)	-	178 (49.7%)	-	
Women	279 (49.6%)	-	180 (50.3%)	-	0.851 ^a^
**Age (years)**					
Men	283 (50.4%)	79.2 (78.0–80.4)	178 (49.7%)	77.0 (75.1–78.9)	0.046 ^b^
Women	279 (49.6%)	81.8 (80.7–83.0)	180 (50.3%)	82.2 (80.8–83.5)	0.719 ^b^
All	562 (100%)	80.5 (79.7–81.4)	358 (100%)	79.6 (78.4–80.8)	0.214 ^b^
**Arrhythmia**					
Yes	32 (5.7%)	-	17 (4.8%)	-	
No	530 (94.3%)	-	341 (95.2%)	-	
All	562 (100%)	-	358 (100%)	-	0.534 ^a^
**HR (bpm)**					
Men	283 (50.4%)	88.6 (86.1–91.1)	178 (49.7%)	84.4 (81.1–87.6)	0.041 ^b^
Women	279 (49.6%)	88.7 (85.7–91.6)	180 (50.3%)	88.6 (85.3–91.9)	0.992 ^b^
All	562 (100%)	88.6 (86.7–90.5)	358 (100%)	86.6 (84.2–88.9)	0.180 ^b^
**SBP (mmHg)**					
Men	283 (50.4%)	141.1 (137.9–144.4)	178 (49.7%)	137.5 (133.4–141.6)	0.173 ^b^
Women	279 (49.6%)	143.0 (139.9–146.1)	180 (50.3%)	141.8 (137.9–145.7)	0.644 ^b^
All	562 (100%)	142.1 (139.8–144.3)	358 (100%)	139.7 (136.8–142.5)	0.197 ^b^
**SBP > 180 mmHg**					
Men	21 (7.4%)	199.9 (191.4–208.5)	9 (5.1%)	198.7 (186.3–211.1)	0.865 ^b^
Women	21 (7.5%)	195.1 (191.4–198.7	11 (6.1%)	197.9 (187.8–208.0)	0.484 ^b^
All	42 (7.5%)	197.5 (193.0–202.0)	20 (5.6%)	198.3 (191.2–205.3)	0.847 ^b^
**DBP (mmHg)**					
Men	283 (50.4%)	79.7 (77.8–81.6)	178 (49.7%)	78.5 (76.2–80.9)	0.447 ^b^
Women	279 (49.6%)	79.4 (77.4–81.5)	180 (50.3%)	77.2 (74.7–79.7)	0.185 ^b^
All	562 (100%)	79.5 (78.2–80.9)	358 (100%)	77.8 (76.1–79.6)	0.134 ^b^
**DBP > 110 mmHg**					
Men	6 (2.1%)	125.8 (114.6–137.1)	6 (3.4%)	120.0 (114.3–125.8)	0.262 ^b^
Women	9 (3.2%)	119.7 (116.2–123.2)	6 (3.3%)	119.3 (112.6–126.0)	0.907 ^b^
All	15 (2.7%)	122.1 (117.8–126.5)	12 (3.4%)	119.7 (116.1–123.3)	0.372 ^b^
**SatO_2_**					
Men	283 (50.4%)	94.4 (93.8–95.0)	178 (49.7%)	94.2 (93.2–95.2)	0.741 ^b^
Women	279 (49.6%)	94.0 (93.3–94.7)	180 (50.3%)	94.3 (93.3–95.2)	0.670 ^b^
All	562 (100%)	94.2 (93.7–94.7)	358 (100%)	94.2 (93.6–94.9)	0.943 ^b^
**SatO_2_ < 85%**					
Men	14 (4.9%)	78.8 (75.0–82.5)	8 (4.5%)	72.6 (65.6–79.6)	0.068 ^b^
Women	18 (6.5%)	77.9 (75.0–80.8)	9 (5.0%)	75.8 (70.2–81.4)	0.421 ^b^
All	32 (5.7%)	78.3 (76.1–80.5)	17 (4.8%)	74.3 (70.3–78.3)	0.052 ^b^
**Priority Code–Access**					
Green	109 (19.4%)	-	103 (28.8%)	-	
Yellow	387 (68.9%)	-	212 (59.2%)	-	
Red	66 (11.7%)	-	43 (12.0%)	-	0.004 ^a^
**Priority Code–Discharge**					
Green	221 (39.3%)	-	121 (33.8%)	-	
Yellow	332 (59.1%)	-	217 (60.6%)	-	
Red	9 (1.6%)	-	20 (5.6%)	-	0.001 ^a^
**Wait time (min)**					
Men	283 (50.4%)	50.6 (43.8–57.3)	178 (49.7%)	60.5 (49.5–71.6)	0.108 ^b^
Women	279 (49.6%)	50.6 (44.1–57.0)	180 (50.3%)	58.8 (49.5–68.0)	0.141 ^b^
All	562 (100%)	50.6 (45.9–55.2)	358 (100%)	59.6 (52.5–66.8)	0.029 ^b^
**Process time (min)**					
Men	283 (50.4%)	578.5 (533.3–623.6)	178 (49.7%)	306.1 (265.9–346.3)	<0.001 ^b^
Women	279 (49.6%)	634.2 (587.2–681.1)	180 (50.3%)	348.8 (304.6–392.9)	<0.001 ^b^
All	562 (100%)	606.1 (573.6–638.7)	358 (100%)	327.6 (297.7–357.4)	<0.001 ^b^
**Total time (min)**					
Men	283 (50.4%)	607.9 (560.3–655.4)	178 (49.7%)	350.9 (312.0–389.8)	<0.001 ^b^
Women	279 (49.6%)	642.9 (593.5–692.3)	180 (50.3%)	402.9 (358.7–447.0)	<0.001 ^b^
All	562 (100%)	625.3 (591.1–659.5)	358 (100%)	377.0 (347.6–406.5)	<0.001 ^b^

HR: Heart rate; SBP: Systolic blood pressure; DBP: Diastolic blood pressure; Sat0_2_: Oxygen saturation. ^a^: χ^2^ test; ^b^: Student’s *t*-test, OBI: brief intensive observation.

**Table 2:** Frequency of principal outcome by group.

	OBI		Non-OBI		*p*
	***N***	***%***	***N***	***%***	
**Death**					
Yes	3	0.53%	3	0.84%	
No	559	99.47%	355	99.16%	0.683 ^b^
**Hospitalization**					
Yes	333	59.25%	245	68.44%	
No	229	40.75%	113	31.56%	**0.005 ^a^**
Transfer *					
Yes	91	16.19%	23	6.42%	
No	471	83.81%	335	93.58%	**<0.001 ^a^**
**Outcomes**					
Hospitalization	333	59.25%	245	68.44%	
Discharge	129	22.95%	83	23.18%	
Transfer *	91	16.19%	23	6.42%	
Voluntary leaving	5	0.89%	4	1.12%	
Hospitalization refuse	1	0.18%	-	-	
Death	3	0.53%	3	0.84%	**<0.001 ^b^**
**Readmission**					
Yes	64	11.4%	35	9.8%	
No	498	88.6%	323	90.2%	0.591 ^a^
**Readmission at 7 days**					
Yes	13	2.31%	12	3.35%	
No	549	97.69%	346	96.65%	0.345 ^a^
**Readmission at 14 days**					
Yes	32	5.69%	21	5.87%	
No	530	94.31%	337	94.13%	0.913 ^a^
**Readmission at 30 days**					
Yes	66	11.74%	40	11.17%	
No	496	88.26%	318	88.83%	0.792 ^a^

* Transfers to other hospital wards or structures. ^a^: χ^2^ test; ^b^: Fisher’s exact test.

**Table 3:** Results of univariate and multivariate logistic regression models. Comparison of favorable (“green” and “yellow” priority codes vs. a “red’” priority code) outcome at discharge.

	OR	95% CI	*p*
*Univariate analysis*			
Non-OBI	1 (reference)	-	
OBI	0.275	0.124–0.611	0.002
*Multivariate analysis*			
OBI (yes vs. no)	0.347	0.130–0.928	0.035
Age (year)	0.971	0.936–1.008	0.122
Sex (male vs. female)	1.332	0.518–3.426	0.626
Arrhythmia (yes vs. no)	1.234	0.246–6.189	0.798
HR (bmp)	1.031	1.012–1.049	0.001
SatO_2_ (%)	0.986	0.931–1.044	0.626
SBP (mmHg)	1.010	0.990–1.030	0.349
DBP (mmHg)	0.984	0.951–1.017	0.330
Wait time (min)	0.981	0.964–0.999	0.041
Process time (min)	0.999	0.995–1.004	0.761
Total time (min)	0.999	0.995–1.003	0.581

HR: Heart Rate; SBP: Systolic blood pressure; DBP: Diastolic blood pressure; SatO_2_: Oxygen saturation.
